# (*E*)-2,3-Bis(4-methoxy­phen­yl)acrylic acid

**DOI:** 10.1107/S1600536809011751

**Published:** 2009-04-02

**Authors:** Banfeng Ruan, Ying Yang, Zhenwei Zhu, Pengcheng Lv, Hailiang Zhu

**Affiliations:** a State Key Laboratory of Pharmaceutical Biotechnology, Nanjing University, Nanjing 210093, People’s Republic of China

## Abstract

In the title mol­ecule, C_17_H_16_O_4_, the angle between the aromatic ring planes is 69.1 (6)°. The crystal structure is stabilized by inter­molecular O—H⋯O hydrogen bonds; mol­ecules related by a centre of symmetry are linked to form inversion dimers.

## Related literature

For the biological properties and synthesis of resveratrol (*trans*-3,4′,5-trihydroxy­stilbene) and its derivatives, see: Huang, Ruan *et al.* (2007 ▶); Huang *et al.* (2008 ▶); Jang *et al.* (1997 ▶); Ruan *et al.* (2006 ▶); Schulze *et al.* (2005 ▶); Shi *et al.* (2005 ▶). For related crystal structures, see: Huang, Li *et al.* (2007 ▶); Stomberg *et al.* (2001 ▶).
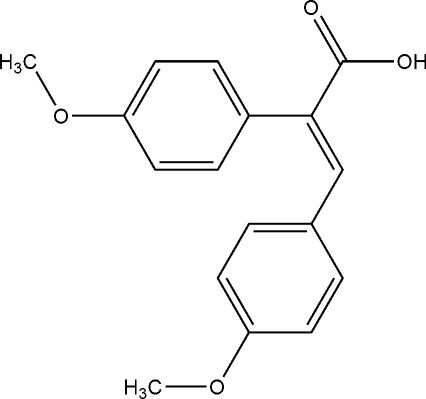


## Experimental

### 

#### Crystal data

C_17_H_16_O_4_
*M* *_r_* = 284.30
Triclinic, 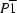

*a* = 5.8690 (12) Å
*b* = 9.1480 (18) Å
*c* = 13.992 (3) Å
α = 83.65 (3)°
β = 85.43 (3)°
γ = 80.92 (3)°
*V* = 735.8 (3) Å^3^
*Z* = 2
Mo *K*α radiation
μ = 0.09 mm^−1^
*T* = 298 K
0.30 × 0.20 × 0.10 mm

#### Data collection

Enraf–Nonius CAD-4 diffractometer
Absorption correction: ψ scan (North *et al.*, 1968 ▶) *T*_min_ = 0.973, *T*_max_ = 0.991
3196 measured reflections
2895 independent reflections
1779 reflections with *I* > 2σ(*I*)
*R*_int_ = 0.027

#### Refinement

*R*[*F* ^2^ > 2σ(*F* ^2^)] = 0.056
*wR*(*F* ^2^) = 0.151
*S* = 1.08
2895 reflections
194 parameters
H-atom parameters constrained
Δρ_max_ = 0.18 e Å^−3^
Δρ_min_ = −0.17 e Å^−3^

### 

Data collection: *CAD-4 Software* (Enraf–Nonius, 1989 ▶); cell refinement: *CAD-4 Software*; data reduction: *XCAD4* (Harms & Wocadlo, 1995 ▶); program(s) used to solve structure: *SHELXS97* (Sheldrick, 2008 ▶); program(s) used to refine structure: *SHELXL97* (Sheldrick, 2008 ▶); molecular graphics: *SHELXTL* (Sheldrick, 2008 ▶); software used to prepare material for publication: *SHELXTL*.

## Supplementary Material

Crystal structure: contains datablocks global, I. DOI: 10.1107/S1600536809011751/wn2320sup1.cif

Structure factors: contains datablocks I. DOI: 10.1107/S1600536809011751/wn2320Isup2.hkl

Additional supplementary materials:  crystallographic information; 3D view; checkCIF report

## Figures and Tables

**Table 1:** Hydrogen-bond geometry (Å, °)

*D*—H⋯*A*	*D*—H	H⋯*A*	*D*⋯*A*	*D*—H⋯*A*
O3—H3⋯O2^i^	0.82	1.80	2.608 (2)	169

Symmetry code: (i) 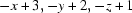.

**Fig. 1.**
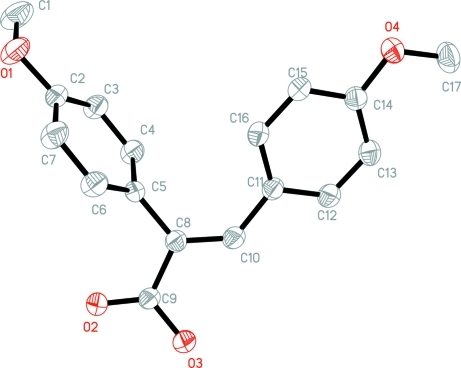
The molecular structure of the title compound, showing 30% probability displacement ellipsoids and the atom-numbering scheme. All H atoms have been omitted.

**Fig. 2.**
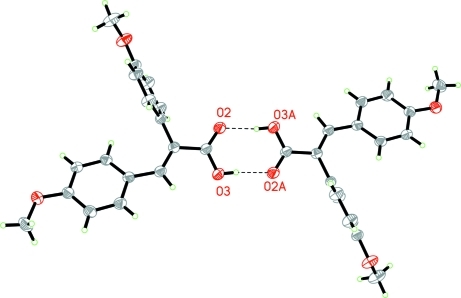
A view of the hydrogen-bonded dimer of the title compound. Dashed lines indicate hydrogen bonds. [Symmetry code: (A) -x + 3, -y + 2, -z + 1]

## supplementary crystallographic information

### Comment

Resveratrol (*trans*-3, 4',5-trihydroxystilbene) and its derivatives have attracted much attention since it was first isolated in 1939, because of their physiological properties and potential therapeutic values (Schulze *et al.*, 2005; Jang *et al.*, 1997). In our laboratory, we have synthesized two series of resveratrol derivatives (Ruan *et al.*, 2006; Huang *et al.*, 2007). As part of an extensive structure-activity relationship (SAR) study on resveratrol derivatives, another series of analogues of resveratrol has been synthesized. One of them, namely the title compound, was obtained as single crystals and its crystal structure determined to establish its configuration.

The crystal structure demonstrated that it had the E configuration (Fig. 1). All bond lengths are within normal ranges and very similar to those in related crystal structures (Stomberg *et al.*, 2001). The torsion angles C5—C8—C10—C11 and C9—C8—C10—C11 are 5.9 (4)° and -176.6 (2)°, respectively. The angle between the aromatic ring planes is 69.1 (6)°. In the crystal structure, molecules related by a centre of symmetry are linked to form dimers *via* intermolecular O—H—O hydrogen bonds (Table 1 and Fig. 2).

### Experimental

2-(4-Methoxyphenyl)acetic acid (1.66 g, 0.01 mol), 4-methoxybenzaldehyde (1.36 g, 0.01 mol) and acetic anhydride (15 ml) were added to a three-necked flask in an icewater bath with stirring. Triethylamine (5 ml) was added dropwise into this solution and the mixture was allowed to react at 100°C for 12 h. After cooling to room temperature, the mixture was slowly poured into 50 ml 10% NaOH solution, yielding a white precipitate. This was collected by vacuum filtration, washed with a large amount of water and dried in air. Colorless single crystals were obtained after a week upon evaporation of a solution of the reaction product in a mixture of ethyl acetate (10 ml) and petroleum ether (5 ml).

### Refinement

All hydrogen atoms were placed in geometrically idealized positions and constrained to ride on their parent atoms, with C—H = 0.93 Å (Csp^2^), 0.96 Å (methyl) and O—H = 0.82 Å. *U*_iso_(H) = 1.2*U*_eq_(Csp^2^), 1.5*U*_eq_(methyl C and hydroxyl O).

### Crystal data

**Table:**

C_17_H_16_O_4_	*Z* = 2
*M**_r_* = 284.30	*F*(000) = 300
Triclinic, *P*1	*D*_x_ = 1.283 Mg m^−^^3^
Hall symbol: -P 1	Mo *K*α radiation, λ = 0.71073 Å
*a* = 5.8690 (12) Å	Cell parameters from 1625 reflections
*b* = 9.1480 (18) Å	θ = 2.2–24.8°
*c* = 13.992 (3) Å	µ = 0.09 mm^−^^1^
α = 83.65 (3)°	*T* = 298 K
β = 85.43 (3)°	Block, colorless
γ = 80.92 (3)°	0.30 × 0.20 × 0.10 mm
*V* = 735.8 (3) Å^3^	

### Data collection

**Table:**

Enraf–Nonius CAD-4 diffractometer	2895 independent reflections
Radiation source: fine-focus sealed tube	1779 reflections with *I* > 2σ(*I*)
graphite	*R*_int_ = 0.027
ω/2θ scans	θ_max_ = 26.0°, θ_min_ = 1.5°
Absorption correction: ψ scan (North *et al.*, 1968)	*h* = 0→7
*T*_min_ = 0.973, *T*_max_ = 0.991	*k* = −11→11
3196 measured reflections	*l* = −17→17

### Refinement

**Table:**

Refinement on *F*^2^	Secondary atom site location: difference Fourier map
Least-squares matrix: full	Hydrogen site location: inferred from neighbouring sites
*R*[*F*^2^ > 2σ(*F*^2^)] = 0.056	H-atom parameters constrained
*wR*(*F*^2^) = 0.151	*w* = 1/[σ^2^(*F*_o_^2^) + (0.0675*P*)^2^ + 0.024*P*] where *P* = (*F*_o_^2^ + 2*F*_c_^2^)/3
*S* = 1.08	(Δ/σ)_max_ < 0.001
2895 reflections	Δρ_max_ = 0.18 e Å^−^^3^
194 parameters	Δρ_min_ = −0.17 e Å^−^^3^
0 restraints	Extinction correction: *SHELXL97* (Sheldrick, 2008), Fc^*^=kFc[1+0.001xFc^2^λ^3^/sin(2θ)]^-1/4^
Primary atom site location: structure-invariant direct methods	Extinction coefficient: 0.044 (7)

### Special details

**Table:**

Geometry. All e.s.d.'s (except the e.s.d. in the dihedral angle between two l.s. planes) are estimated using the full covariance matrix. The cell e.s.d.'s are taken into account individually in the estimation of e.s.d.'s in distances, angles and torsion angles; correlations between e.s.d.'s in cell parameters are only used when they are defined by crystal symmetry. An approximate (isotropic) treatment of cell e.s.d.'s is used for estimating e.s.d.'s involving l.s. planes.
Refinement. Refinement of *F*^2^ against ALL reflections. The weighted *R*-factor *wR* and goodness of fit *S* are based on *F*^2^, conventional *R*-factors *R* are based on *F*, with *F* set to zero for negative *F*^2^. The threshold expression of *F*^2^ > σ(*F*^2^) is used only for calculating *R*-factors(gt) *etc*. and is not relevant to the choice of reflections for refinement. *R*-factors based on *F*^2^ are statistically about twice as large as those based on *F*, and *R*- factors based on ALL data will be even larger.

### Fractional atomic coordinates and isotropic or equivalent isotropic displacement parameters (Å^2^)

**Table:**

	*x*	*y*	*z*	*U*_iso_*/*U*_eq_	
C1	0.7983 (6)	1.3447 (4)	−0.0150 (2)	0.0938 (11)	
H1A	0.6774	1.2848	−0.0132	0.141*	
H1B	0.8108	1.3993	−0.0774	0.141*	
H1C	0.7629	1.4130	0.0333	0.141*	
C2	1.0269 (4)	1.1661 (3)	0.08912 (15)	0.0505 (6)	
C3	0.8532 (4)	1.1641 (3)	0.16030 (17)	0.0565 (6)	
H3A	0.7103	1.2225	0.1513	0.068*	
C4	0.8905 (4)	1.0754 (3)	0.24524 (17)	0.0548 (6)	
H4	0.7705	1.0752	0.2929	0.066*	
C5	1.0983 (4)	0.9870 (2)	0.26254 (15)	0.0423 (5)	
C6	1.2741 (4)	0.9919 (3)	0.19039 (16)	0.0553 (6)	
H6	1.4182	0.9357	0.2001	0.066*	
C7	1.2382 (4)	1.0786 (3)	0.10484 (17)	0.0621 (7)	
H7	1.3573	1.0786	0.0568	0.074*	
C8	1.1324 (4)	0.8926 (2)	0.35563 (15)	0.0462 (6)	
C9	1.2916 (4)	0.9385 (3)	0.41980 (16)	0.0495 (6)	
C10	1.0275 (4)	0.7750 (2)	0.38730 (16)	0.0500 (6)	
H10	1.0582	0.7349	0.4498	0.060*	
C11	0.8736 (4)	0.6996 (2)	0.34031 (15)	0.0466 (6)	
C12	0.7562 (4)	0.5977 (3)	0.39652 (17)	0.0563 (7)	
H12	0.7770	0.5821	0.4623	0.068*	
C13	0.6106 (4)	0.5188 (3)	0.35884 (17)	0.0577 (7)	
H13	0.5354	0.4505	0.3985	0.069*	
C14	0.5767 (4)	0.5414 (2)	0.26176 (17)	0.0495 (6)	
C15	0.6987 (4)	0.6386 (3)	0.20313 (16)	0.0550 (6)	
H15	0.6826	0.6504	0.1370	0.066*	
C16	0.8418 (4)	0.7169 (2)	0.24152 (16)	0.0521 (6)	
H16	0.9198	0.7831	0.2013	0.063*	
C17	0.2995 (5)	0.3745 (3)	0.2726 (2)	0.0746 (8)	
H17A	0.3998	0.2948	0.3053	0.112*	
H17B	0.2067	0.3345	0.2315	0.112*	
H17C	0.2009	0.4283	0.3192	0.112*	
O1	1.0108 (3)	1.2517 (2)	0.00292 (12)	0.0753 (6)	
O2	1.3849 (3)	1.04878 (19)	0.39719 (11)	0.0674 (6)	
O3	1.3251 (3)	0.85636 (19)	0.50110 (11)	0.0691 (6)	
H3	1.4025	0.8958	0.5341	0.104*	
O4	0.4348 (3)	0.47241 (18)	0.21611 (12)	0.0641 (5)	

### Atomic displacement parameters (Å^2^)

**Table:**

	*U*^11^	*U*^22^	*U*^33^	*U*^12^	*U*^13^	*U*^23^
C1	0.091 (2)	0.104 (2)	0.074 (2)	0.007 (2)	−0.0214 (18)	0.0273 (18)
C2	0.0525 (15)	0.0561 (14)	0.0426 (12)	−0.0126 (12)	−0.0038 (11)	0.0029 (10)
C3	0.0391 (13)	0.0660 (16)	0.0582 (14)	0.0010 (12)	−0.0025 (11)	0.0085 (12)
C4	0.0350 (12)	0.0696 (16)	0.0543 (14)	−0.0040 (11)	0.0048 (10)	0.0067 (12)
C5	0.0397 (12)	0.0455 (12)	0.0429 (12)	−0.0120 (10)	−0.0034 (9)	−0.0004 (9)
C6	0.0366 (13)	0.0690 (16)	0.0543 (14)	0.0024 (11)	0.0015 (11)	0.0018 (12)
C7	0.0468 (15)	0.0817 (18)	0.0506 (14)	−0.0023 (13)	0.0095 (12)	0.0054 (13)
C8	0.0419 (13)	0.0483 (13)	0.0475 (12)	−0.0076 (10)	−0.0032 (10)	0.0010 (10)
C9	0.0472 (13)	0.0552 (14)	0.0451 (13)	−0.0108 (11)	−0.0061 (11)	0.0061 (11)
C10	0.0515 (14)	0.0544 (14)	0.0432 (12)	−0.0101 (11)	−0.0037 (11)	0.0034 (10)
C11	0.0437 (13)	0.0481 (13)	0.0465 (13)	−0.0074 (10)	−0.0032 (10)	0.0030 (10)
C12	0.0595 (16)	0.0638 (16)	0.0461 (13)	−0.0183 (13)	−0.0028 (11)	0.0053 (11)
C13	0.0527 (15)	0.0620 (15)	0.0595 (15)	−0.0223 (12)	0.0017 (12)	0.0050 (12)
C14	0.0434 (13)	0.0456 (13)	0.0588 (14)	−0.0050 (11)	−0.0071 (11)	−0.0009 (11)
C15	0.0662 (16)	0.0519 (14)	0.0475 (13)	−0.0145 (12)	−0.0108 (12)	0.0059 (11)
C16	0.0544 (14)	0.0522 (14)	0.0502 (14)	−0.0178 (12)	−0.0027 (11)	0.0064 (11)
C17	0.0668 (18)	0.0648 (17)	0.097 (2)	−0.0284 (15)	−0.0050 (16)	−0.0028 (15)
O1	0.0737 (13)	0.0920 (14)	0.0513 (10)	−0.0045 (11)	−0.0040 (9)	0.0201 (9)
O2	0.0759 (12)	0.0728 (12)	0.0594 (11)	−0.0376 (10)	−0.0217 (9)	0.0176 (9)
O3	0.0865 (14)	0.0735 (12)	0.0535 (10)	−0.0355 (10)	−0.0255 (9)	0.0150 (9)
O4	0.0639 (11)	0.0610 (11)	0.0720 (11)	−0.0240 (9)	−0.0171 (9)	0.0030 (9)

### Geometric parameters (Å, °)

**Table:**

C1—O1	1.418 (3)	C9—O3	1.303 (2)
C1—H1A	0.9600	C10—C11	1.455 (3)
C1—H1B	0.9600	C10—H10	0.9300
C1—H1C	0.9600	C11—C12	1.384 (3)
C2—O1	1.364 (3)	C11—C16	1.398 (3)
C2—C3	1.368 (3)	C12—C13	1.371 (3)
C2—C7	1.385 (3)	C12—H12	0.9300
C3—C4	1.375 (3)	C13—C14	1.377 (3)
C3—H3A	0.9300	C13—H13	0.9300
C4—C5	1.376 (3)	C14—O4	1.356 (3)
C4—H4	0.9300	C14—C15	1.386 (3)
C5—C6	1.387 (3)	C15—C16	1.362 (3)
C5—C8	1.490 (3)	C15—H15	0.9300
C6—C7	1.372 (3)	C16—H16	0.9300
C6—H6	0.9300	C17—O4	1.423 (3)
C7—H7	0.9300	C17—H17A	0.9600
C8—C10	1.339 (3)	C17—H17B	0.9600
C8—C9	1.482 (3)	C17—H17C	0.9600
C9—O2	1.222 (3)	O3—H3	0.8200
			
O1—C1—H1A	109.5	C8—C10—C11	130.8 (2)
O1—C1—H1B	109.5	C8—C10—H10	114.6
H1A—C1—H1B	109.5	C11—C10—H10	114.6
O1—C1—H1C	109.5	C12—C11—C16	116.9 (2)
H1A—C1—H1C	109.5	C12—C11—C10	117.9 (2)
H1B—C1—H1C	109.5	C16—C11—C10	125.1 (2)
O1—C2—C3	124.8 (2)	C13—C12—C11	122.4 (2)
O1—C2—C7	116.4 (2)	C13—C12—H12	118.8
C3—C2—C7	118.8 (2)	C11—C12—H12	118.8
C2—C3—C4	119.8 (2)	C12—C13—C14	119.5 (2)
C2—C3—H3A	120.1	C12—C13—H13	120.3
C4—C3—H3A	120.1	C14—C13—H13	120.3
C3—C4—C5	122.6 (2)	O4—C14—C13	125.2 (2)
C3—C4—H4	118.7	O4—C14—C15	115.5 (2)
C5—C4—H4	118.7	C13—C14—C15	119.3 (2)
C4—C5—C6	117.04 (19)	C16—C15—C14	120.6 (2)
C4—C5—C8	121.03 (19)	C16—C15—H15	119.7
C6—C5—C8	121.9 (2)	C14—C15—H15	119.7
C7—C6—C5	121.0 (2)	C15—C16—C11	121.2 (2)
C7—C6—H6	119.5	C15—C16—H16	119.4
C5—C6—H6	119.5	C11—C16—H16	119.4
C6—C7—C2	120.8 (2)	O4—C17—H17A	109.5
C6—C7—H7	119.6	O4—C17—H17B	109.5
C2—C7—H7	119.6	H17A—C17—H17B	109.5
C10—C8—C9	117.8 (2)	O4—C17—H17C	109.5
C10—C8—C5	126.2 (2)	H17A—C17—H17C	109.5
C9—C8—C5	115.95 (18)	H17B—C17—H17C	109.5
O2—C9—O3	122.1 (2)	C2—O1—C1	118.0 (2)
O2—C9—C8	121.2 (2)	C9—O3—H3	109.5
O3—C9—C8	116.7 (2)	C14—O4—C17	118.3 (2)
			
O1—C2—C3—C4	−178.7 (2)	C9—C8—C10—C11	−176.6 (2)
C7—C2—C3—C4	−0.3 (4)	C5—C8—C10—C11	5.9 (4)
C2—C3—C4—C5	0.1 (4)	C8—C10—C11—C12	−168.2 (2)
C3—C4—C5—C6	0.9 (4)	C8—C10—C11—C16	14.8 (4)
C3—C4—C5—C8	179.8 (2)	C16—C11—C12—C13	−1.5 (4)
C4—C5—C6—C7	−1.7 (4)	C10—C11—C12—C13	−178.8 (2)
C8—C5—C6—C7	179.4 (2)	C11—C12—C13—C14	−0.5 (4)
C5—C6—C7—C2	1.5 (4)	C12—C13—C14—O4	−178.9 (2)
O1—C2—C7—C6	178.1 (2)	C12—C13—C14—C15	2.9 (4)
C3—C2—C7—C6	−0.4 (4)	O4—C14—C15—C16	178.3 (2)
C4—C5—C8—C10	66.9 (3)	C13—C14—C15—C16	−3.3 (4)
C6—C5—C8—C10	−114.2 (3)	C14—C15—C16—C11	1.3 (4)
C4—C5—C8—C9	−110.7 (2)	C12—C11—C16—C15	1.1 (3)
C6—C5—C8—C9	68.2 (3)	C10—C11—C16—C15	178.2 (2)
C10—C8—C9—O2	−176.7 (2)	C3—C2—O1—C1	−0.5 (4)
C5—C8—C9—O2	1.1 (3)	C7—C2—O1—C1	−178.9 (2)
C10—C8—C9—O3	2.9 (3)	C13—C14—O4—C17	3.6 (3)
C5—C8—C9—O3	−179.4 (2)	C15—C14—O4—C17	−178.2 (2)

### Hydrogen-bond geometry (Å, °)

**Table:**

*D*—H···*A*	*D*—H	H···*A*	*D*···*A*	*D*—H···*A*
O3—H3···O2^i^	0.82	1.80	2.608 (2)	169

Symmetry codes: (i) −*x*+3, −*y*+2, −*z*+1.
